# Investigating the relationship between women’s experience of intimate partner violence and utilization of maternal healthcare services in India

**DOI:** 10.1038/s41598-021-89688-1

**Published:** 2021-05-27

**Authors:** Pintu Paul, Dinabandhu Mondal

**Affiliations:** Centre for the Study of Regional Development, School of Social Sciences, Jawaharlal Nehru University, New Delhi, 110067 India

**Keywords:** Health policy, Health services, Public health

## Abstract

The utilization of maternal healthcare services is a key measure to reduce the incidence of maternal mortality. This study aims to examine the relationship between women’s exposure to intimate partner violence (IPV) and the utilization of maternal healthcare services, using a large-scale nationally representative data among Indian women. Data for this study were drawn from the fourth round of the National Family Health Survey (NFHS-4), which is collected during 2015–2016. In order to analyze, we utilized 24,882 currently married women aged 15–49 years who had at least one living child in the past five years preceding the survey. Women’s experience of IPV, which is manifested in various forms of physical, emotional, and/or sexual violence perpetrated by the partner, was considered as the key explanatory variable. Adequate antenatal care (ANC) [four or more ANC visits], delivery assistance by the skilled health provider, and postnatal care (PNC) within two days of delivery were used as outcome variables for assessing the utilization of maternal healthcare services. Descriptive statistics, cross-tabulation, Pearson’s chi-square test, and bivariate and multivariate logistic regression models were used in this study. Approximately 26% of the sample women (currently married) experienced any form of IPV in the past year. Bivariate analyses show that the utilization of all three components of maternal healthcare services was lower among women who experienced physical, sexual, or emotional violence, as compared to those who did not face any violence perpetrated by the partner. Multivariate analysis indicates that women’s exposure to IPV was significantly associated with a lower likelihood of adequate ANC utilization (Adjusted Odds Ratio [OR]: 0.90, 95% CI 0.84–0.97), even after controlling for socio-demographic characteristics. However, IPV had no significant relationship with skilled delivery assistance and unexpectedly a positive association with PNC usage (Adjusted OR: 1.09, 95% CI 1.02–1.16) in the adjusted analysis. Our study suggests formulating strategies toward the prevention of husband-perpetrated violence against women and targeting women who experienced spousal violence to improve their utilization of maternal healthcare services.

## Introduction

Violence against women is an important public health concern and an outright violation of human rights and gender equality^1–3^. It is recognized as a worldwide problem of ‘epidemic proportion’ by the World Health Organization (WHO)^4^. The United Nations Declaration on the ‘Elimination of Violence Against Women’ describes violence against women as *“any act of gender-based violence that results in, or is likely to result in, physical, sexual or mental harm or suffering to women, including threats of such acts, coercion or arbitrary deprivation of liberty, whether occurring in public or in private life”*^3^. Intimate partner violence (IPV) is the most common form of violence against women^5^. IPV is abuse or violent action between two individuals in a relationship that is manifested in an intricate form of physical aggression, sexual coercion, psychological abuse, and controlling behaviors^6,7^.

Domestic violence is an unfortunate reality and occurs in almost all countries across the world. Women of all ages, irrespective of socio-cultural and religious identities, geographic boundaries, and economic status experience domestic violence. It is estimated that approximately one in every third woman (35%) worldwide experience domestic violence during their lifetime. Much of the violence occurs within an intimate relationship, with around 30% of women experienced physical and/or sexual violence by their intimate partner^4^. According to the WHO multi-country study on domestic violence and women’s health, the proportion of women subject to physical or sexual abuse considerably varies across the countries, ranging from 15% in Japan to 71% in Ethiopia^8^. In India, the prevalence of IPV remains unacceptably high as the recent National Family Health Survey (NFHS-4) reports that around 27% of women reported experiences of physical violence, 13% experienced emotional violence, and 6% faced sexual abuse by the partner during 2015–2016^9^.

Gender inequality is a key driver of domestic violence against women worldwide^4^. Despite several interventions by the Indian government, such as recognition of domestic violence as a criminal offense under ‘Indian Penal Code 498-A’ and enactment of ‘Protection of Women from Domestic Violence Act 2005’ (PWDVA) to protect women, violence against women and girls nevertheless remains widespread and a challenging concern in India. It is deeply embedded in the patriarchal family structure and rigid socio-cultural norms of society^10^. In a patriarchal society, men are often placed in a higher level of social order and family structure, and they control over women in several ways^11^. In fact, there is a persistent belief that husbands have the right to control wives’ behavior and even beat their wives in order to educate or punish them^1,12^.

The experience of violence has far-reaching consequences on the victims. It has a multitude of negative effects on women’s physical as well as psychological health^13,14^. Studies have observed that women’s exposure to violence is associated with physical injury and mental health problems, including depression^15–17^, anxiety^18^, suicide attempts^19^, alcoholism, and post-traumatic stress disorders^20,21^. Domestic violence increases the risks of adverse pregnancy outcomes, obstetric complications, childhood morbidity, and neonatal and child mortality^22–24^.

Several studies have established a strong link between IPV and women’s reproductive health status^25–27^. A growing body of literature conducted in Bangladesh^28^, Ethiopia^29^, Timor-Leste^30^, and Mozambique^31^ found that women’s experience of IPV is associated with lower use of antenatal care (ANC) and delivery assistance by a skilled health provider. There are also studies indicating that experience of violence is linked to delayed entry into ANC^32,33^. Exposure to violence during pregnancy is harmful to the mother and fetus growth, as not initiating and missing ANC may result in adverse birth outcomes, for instance, preterm delivery and low birth weight^34^.

Garcia-Morena et al.^4^ documented the pathways of how IPV does instigate psychological trauma, fear, and a dearth of control/autonomy among women that may limit sexual and reproductive control and access to healthcare and eventually result in poor family planning, maternal healthcare, and birth outcomes. This is relevant to India, where 31% of ever-married women experienced husband-perpetrated physical, emotional, and/or sexual violence^9^. Moreover, the country has accounted for a large number of maternal deaths (35,000 deaths in 2017)^35^. Although significant improvement has been witnessed in reducing maternal mortality in the last two decades, the maternal mortality ratio (MMR) of this country is still one of the highest worldwide, 122 per 100,000 live births in 2015–2017^36^. India has to go a long way to achieve the target of Sustainable Development Goal (SDG) of MMR below 70 per 100,000 live births by 2030. Despite recognition of the contributing role of maternal care services for reducing the health risks for mothers and babies, the coverage of ANC and safe delivery care remains unsatisfactory in India. Almost half of the women did not complete the minimum of four ANC visits and 19% of births were delivered without skilled assistance in 2015–2016 as recommended by the WHO for safe pregnancy and delivery outcomes^9^.

Although several previous studies have examined socioeconomic, demographic, and accessibility-related risk factors for low coverages of maternity care^37–40^, the influence of IPV on access to reproductive health services has gained little attention in India. In the backdrop of a higher incidence of violence against women and maternal mortality, it is of paramount importance to explore the link between the experience of violence and utilization of maternity care services. It would provide a new perspective on the design of policy interventions to meet the country’s maternal health care needs. The present study therefore aims to investigate the relationship between women’s exposure to IPV and the utilization of maternal healthcare services among currently married women in India using the most recent large-scale sample survey. In this study, we hypothesized that women’s exposure to IPV is associated with lower use of maternal healthcare services.

## Methods

### Data source

Data were drawn from the 2015–2016 National Family Health Survey (NFHS-4) which is the latest Demographic and Health Survey (DHS) in India. The NFHS-4 is a nationally representative large sample survey carried out under the direction of the Ministry of Health and Family Welfare (MoHFW), Government of India with assistance from the International Institute for Population Sciences (IIPS), Mumbai. The survey covered 699,686 women aged 15–49 years with a response rate of 97%, conducted across all the states and union territories (UTs) of the country. The key purpose of this survey was to provide up-to-date and reliable information on population and health: reproductive health status, family planning, utilization of maternal health care services, child immunization services, nutritional status, breastfeeding practices, childhood morbidity and mortality, domestic violence, women’s empowerment, among others. A stratified two-stage sampling design was adopted for the collection of samples. Based on the 2011 Indian Census enumeration, 28,586 clusters of areas—8397 in urban, 20,059 in rural areas, and 130 in slums were selected in the first stage. For the selection of these clusters, the approach of probability proportional to size (PPS) was used. In the second stage, a complete household mapping and the listing was performed in the chosen clusters and 22 households were selected systematically from the household listing in each cluster. The national report of NFHS-4 provides a detailed description of the sampling design and survey protocol^9^.

### Study design and sample size

**Figure 1:**
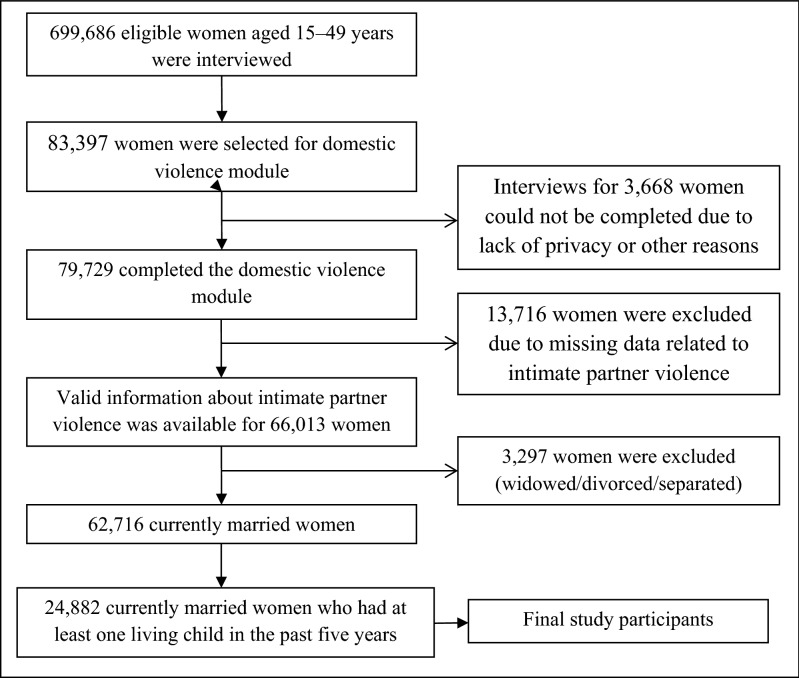
Selection of study participants, NFHS-4 (2015–2016).

We adopted a cross-sectional study design using the most recent round of NFHS for the analyses of this study. The domestic violence module was only applied to one eligible woman per household randomly selected in compliance with the WHO’s ethical requirements for research on domestic violence^41^. In total, 83,397 women were selected in the violence module of this survey. However, 79,729 women completed the interview regarding domestic violence. The violence module for 3668 eligible women could not be completed due to privacy issues or other reasons^9^. A total number of 66,013 ever-married women had complete and valid information about IPV and 13,716 non-married women were excluded because they were not eligible or did not participate in IPV-related questions. Among them, 24,882 currently married women who gave birth to a living child in the past five years constitute the final study participants in this research. All statistical analyses of this study were performed with these 24,882 women who had valid information about IPV and other variables of interest (Fig. 1).

### Outcome variables

The outcome measure of this study is the utilization of maternal healthcare services among currently married women aged 15–49 years who had at least one living child in the past five years before the survey. Maternal healthcare utilization has three main components—Antenatal care (ANC), delivery care, and postnatal care (PNC). Three important indicators were used to assess these components. ANC of the women conceived as the primary care during pregnancy is generally estimated by the number of ANC visits and the timing of the first ANC visit. At least four ANC visits during pregnancy, considered as the adequate number of visits, were taken as an indicator of ANC^42^. Delivery attended by a trained person or in an institution is considered as ‘safe delivery’ as per the WHO standards^43^. Delivery by a skilled person was defined as whether the delivery was assisted by a doctor, ANM/nurse/midwife, or other health personnel. PNC of the mother includes treatment of complications that might have occurred after the delivery along with services like counseling on family planning, breastfeeding practices, nutrition, management of neonatal hypothermia, etc. PNC within two days of delivery was considered as an indicator of postpartum care in this study as per the recommendation of the Ministry of Health and Family Welfare (MoHFW), Government of India^9^. All three indicators—four or more ANC visits (adequate ANC), delivery assistance by the skilled health provider, and PNC within two days of delivery were dichotomized into “yes” (coded as ‘1’) and “no” (coded as ‘0’) for the purpose of this study.

### Explanatory variables

Women’s exposure to IPV in the past year is the key explanatory variable in this study. In the domestic violence module of the NHFS-4, women were asked a series of questions to extract information related to various forms of violence committed by their current husband or most recent husband. Three forms of IPV—physical, emotional, and sexual violence—have been recognized from the violence module. Physical violence is composed of seven questions in which women were asked whether her husband has *(i) “pushed, shook, or thrown something at her” (ii) “slapped her”, (iii) “twisted her arm or pulled her hair”, (iv) “punched her with his fist or with something that could hurt her”, (v) “kicked, dragged, or beaten her up”, (vi) “tried to choke or burn her on purpose”, or (vii) “threaten or attacked with a knife, gun, or any other weapon”* in the past 12 months. Concerning sexual violence, women were asked three questions, whether being (*i) “physically forced to have sexual intercourse”, (ii) “physically forced to perform any other sexual acts” or (iii) “forced with threats or in any other way to perform sexual acts when she did not want to”* in the past 12 months. Likewise, women’s exposure to emotional violence committed by the husband was assessed by three questions, whether her husband has (*i) “humiliated her in front of others”, (iii) “threatened her to hurt or harm”, or (iii) “insulted or made her feel bad about herself”* in the past 12 months. A detailed description of questions and data collection procedures related to violence against women is provided in the national report of NFHS-4^9^. Using this information, each type of violence was aggregated into a variable reflecting three specific forms of IPV, namely physical, sexual, and emotional violence. Furthermore, a dichotomous variable representing any form of IPV was generated as to whether the currently married women being subjected to any kind of violence committed by the husband in the past year.

### Confounders

A wide range of socio-economic and demographic variables was included as confounders that have a potential influence on the utilization of maternal healthcare services. The selection of confounding variables is based on previous research on domestic violence and reproductive healthcare^25–32^. These selected variables include the place of residence (urban, rural), caste (Scheduled Caste [SC]/Scheduled Tribe [ST], Other Backward Class [OBC], others [forward caste]), religion (Hindu, Muslim, others), women’s age (15–24, 25–34, 35–49 years), age at marriage (< 18, ≥ 18 years), parity (1, 2–3, ≥ 4), educational level of women and partners (no education, primary, secondary, higher), women’s employment status (employed, unemployed), household size (1–4, 5–6, ≥ 7), wealth index (poorest, poorer, middle, richer, richest), decision-making on healthcare (wife alone, wife and husband together, husband alone and others), access to mass media (no, yes), and region (north, central, east, northeast, west, south).

It is worthwhile to mention that the wealth index is an indicator of the household’s standard of living. Household wealth quintile was assessed from the ownership of consumer goods (e.g., television, bicycle, car, etc.) and household characteristics (e.g., sources of drinking water, sanitation facilities, and flooring materials). The NFHS-4 generated wealth scores from those household assets for each individual using principal component analysis and classified them into five quintiles, each representing 20% of the respondents, between 1 (poorest) and 5 (richest). Decision-making on healthcare was assessed by a question, *“who usually decides on healthcare”*, asked to the women during the survey, and the responses were categorized as wife alone, wife and partner together, and partner alone or others. Women’s exposure to mass media was assessed by the frequency of reading newspapers and magazines, listening to the radio, and watching television and then divided into two groups: ‘no’ when none of the three media accessed and ‘yes’ when at least one media was accessible. Furthermore, a region variable was developed to understand the regional variations in the utilization of maternal health care services as apparent in several previous studies. We followed the regional categorization of India prepared by the NFHS-4 which is principally based on the geographical contiguity and cultural settings^9^.

### Analytical strategies

Descriptive statistical analyses were carried out to describe the distribution of study participants. To determine the utilization of maternal healthcare services (at least four ANC visits, skilled birth assistance, and PNC within two days of delivery) by the explanatory variables, bivariate percentages were calculated, and the differences were later tested by Pearson’s chi-square statistic. The sample weight was used to estimate the percentage distribution in univariate and bivariate analyses. Finally, bivariate and multivariate binary logistic regression models were employed to examine the unadjusted and adjusted associations between women’s exposure to IPV and the utilization of maternal healthcare services. The results of regression models were presented by the unadjusted and adjusted odds ratios (ORs) with 95% confidence intervals (CIs). Significance levels were set at *p* ≤ 0.05 and *p* ≤ 0.01. Data management and analysis were performed using STATA software (version 14).

### Ethics declaration

The Demographic Health Surveys (DHS) Program provided approval to access the dataset. The NFHS-4 survey protocol was reviewed and approved by the ICF International Institutional Review Board (IRB) and the International Institute for Population Sciences (IIPS). Further separate approval for conducting this research was not required since data are freely available in the public domain.

## Results

### Background characteristics of the respondents

Of 24,882 participants, the majority lived in rural areas (71%) and believed in Hinduism (73%). Slightly over two-fifth (42%) belonged to OBC, followed by SC/ST group (37%). About 58% of women were between the ages of 25–34 years. Over one-third (36%) of women got married before reaching 18 years. Just over half (51%) had parity between two and three. More than one-quarter of women (27%) had no formal education, while around 16% of the husbands had no education. Only around 17% of the respondents were employed at the time of the survey. About 36% of the participants were residing in large households (seven or more household members). The distribution of study participants also varied significantly across wealth quintiles, ranging from 22% in the poorest wealth group to 18% in the richest wealth group. Nearly two-thirds of the respondents (65%) reported that wife and husband jointly generally make the decisions on healthcare. Nearly one in every four women (24%) had no access to mass media (no access to newspapers/magazines, radio, or television). The highest percentage of the respondents resided in the central region (24%), followed by the north (21%) and the east (20%) regions (Table 1).

**Table 1:** Utilization of maternal healthcare services by socio-demographic characteristics and various forms of intimate partner violence (IPV) among currently married women aged 15–49 years who had at least one living child in the past five years before the survey, India, NFHS-4 (*n* = 24,882).

Variables	Frequency (percentage)	Adequate ANC [four or more ANC visits] (%)	Delivery assistance by skilled health provider (%)	PNC within two days of delivery (%)
**Place of residence**				
Urban	6549 (29.2)	66.2	91.1	74.1
Rural	18,333 (70.8)	45.5	77.9	60.7
*p* value		< 0.001	< 0.001	< 0.001
**Caste**				
SC/ST	9545 (37.0)	46.5	75.5	59.5
OBC	9466 (41.9)	48.3	84.3	65.6
Forward caste	4583 (21.2)	62.6	87.2	71.7
*p* value		< 0.001	< 0.001	< 0.001
**Religion**				
Hindu	17,924 (72.6)	51.0	84.1	65.9
Muslim	3970 (16.7)	53.4	77.8	62.4
Others	2988 (10.7)	52.2	72.2	59.1
*p* value		< 0.001	< 0.001	< 0.001
**Women's age (years)**				
15–24	6985 (31.1)	51.7	84.8	65.7
25–34	15,016 (57.6)	52.6	82.1	65.3
35–49	2881 (11.3)	45.6	71.9	57.8
*p* value		< 0.001	< 0.001	< 0.001
**Age at marriage (years)**				
< 18	8717 (35.8)	39.2	75.3	56.9
≥ 18	15,760 (64.2)	58.8	85.9	69.5
*p* value		< 0.001	< 0.001	< 0.001
**Parity**				
1	7321 (32.6)	62.3	91.0	72.7
2–3	12,991 (50.7)	52.4	82.2	65.0
≥ 4	4570 (16.7)	28.0	62.5	47.7
*p* value		< 0.001	< 0.001	< 0.001
**Women's education**				
No education	7142 (26.9)	29.8	65.9	51.1
Primary	3438 (13.1)	45.2	76.6	59.4
Secondary	11,586 (47.8)	59.6	88.4	69.9
Higher	2716 (12.2)	75.1	96.4	79.3
*p* value		< 0.001	< 0.001	< 0.001
**Partner's education**				
No education	4323 (15.9)	30.4	63.9	48.0
Primary	3596 (13.9)	42.8	74.5	58.5
Secondary	13,530 (55.3)	55.3	85.3	67.6
Higher	3372 (14.9)	68.9	94.6	76.9
*p* value		< 0.001	< 0.001	< 0.001
**Women’s employment status**				
Unemployed	20,420 (83.2)	52.2	83.0	64.9
Employed	4462 (16.8)	48.5	75.7	63.0
*p* value		< 0.001	< 0.001	0.015
**Household size**				
1–4	8408 (28.4)	57.3	85.3	67.9
5–6	9947 (35.9)	52.0	80.5	64.2
≥ 7	6527 (35.7)	46.5	80.2	62.3
*p* value		< 0.001	< 0.001	< 0.001
**Wealth index**				
Poorest	6060 (22.2)	25.5	63.2	47.7
Poorer	5634 (21.4)	41.2	76.1	56.8
Middle	5046 (19.8)	57.0	86.1	68.5
Richer	4343 (18.5)	66.5	91.7	73.2
Richest	3799 (18.1)	74.8	96.4	81.2
*p* value		< 0.001	< 0.001	< 0.001
**Decision-making on healthcare**				
Wife alone	2231 (9.0)	54.0	82.8	65.6
Wife & Husband	16,257 (64.7)	54.0	82.1	65.8
Husband alone/others	6394 (26.3)	44.6	80.7	61.2
*p* value		< 0.001	0.206	< 0.001
**Access to mass media**				
No	6344 (23.9)	25.3	64.8	47.9
Yes	18,538 (76.1)	59.9	87.1	69.8
*p* value		< 0.001	< 0.001	< 0.001
**Region**				
North	5164 (20.7)	59.3	87.4	72.6
Central	6128 (24.4)	34.0	76.5	59.8
East	4906 (20.1)	38.4	77.3	58.2
Northeast	3574 (14.2)	50.0	71.4	54.1
West	2229 (9.1)	74.9	89.8	74.2
South	2881 (11.4)	81.5	97.2	76.8
*p* value		< 0.001	< 0.001	< 0.001
**Physical violence**				
No	19,208 (77.8)	54.5	83.3	65.9
Yes	5674 (22.2)	41.4	76.3	60.0
*p* value		< 0.001	< 0.001	< 0.001
**Sexual violence**				
No	23,412 (93.9)	52.5	82.3	65.0
Yes	1470 (6.1)	37.4	73.9	58.5
*p* value		< 0.001	< 0.001	< 0.001
**Emotional violence**				
No	22,287 (89.7)	52.4	82.4	65.3
Yes	2595 (10.3)	44.3	76.5	58.8
*p* value		< 0.001	< 0.001	< 0.001
**Any form of violence**				
No	18,272 (74.1)	54.7	83.6	66.1
Yes	6610 (25.9)	42.6	76.6	60.3
*p* value		< 0.001	< 0.001	< 0.001

*p* values represent the level of significance of Pearson’s chi-square statistic.

### Prevalence of intimate partner violence

About 22% of the study participants reported experience of physical violence, 6% experienced sexual violence, and 10% were emotionally abused by the partner in the past year. Moreover, over one-fourth (26%) reported that they had experienced any form of IPV in the past year (Table 1).

### Utilization of maternal healthcare services by explanatory variables

Bivariate associations show considerable differences in the use of maternal healthcare services (adequate ANC visits, delivery assistance by the skilled health provider, and PNC within two days of delivery) by socio-demographic background characteristics. The use of maternal healthcare services was higher among women living in urban areas as compared to their rural counterparts. Among caste groups, women who belonged to marginalized caste groups (SC/ST) had received less maternity care than the forward caste women. Respondents who affiliated with a Hindu religion were more likely to deliver by the skilled health personnel and receive PNC check-ups, while Muslim women slightly in an advantageous position in receiving adequate ANC than Hindu women. The percentage of receiving maternal healthcare services was lower among older women (aged 35–49 years) and those who married before 18 years. Similarly, women with parity above six, illiterate, employed, and those residing in large households received less maternity care services. The utilization of maternal healthcare considerably varied across wealth groups: lowest in the poorest group to highest in the richest wealth group. The percentages of adequate ANC and PNC usage were significantly lower among those respondents whose husbands alone or other family members make decisions on healthcare. Women with access to mass media received a higher proportion of maternal healthcare services. The results also show that the utilization of maternal healthcare facilities differed considerably across geographical regions. The usage of adequate ANC was highest in the south, while the lowest ANC utilization was observed in the east region. The proportion of women who delivered by the skilled health provider and received PNC was higher in the south, whereas low use of skilled delivery assistance and PNC service was found in the northeast region. The utilization of maternal healthcare was lower among those women who experienced physical, sexual, or emotional violence, as compared to those who did not face any violence perpetrated by the partner (Table 1).

### The relationship between intimate partner violence and utilization of maternal healthcare services

Table 2 presents the results of unadjusted and adjusted logistic regression models investigating the relationship between women’s exposure to IPV and the utilization of maternal healthcare services. Adjusted models were controlled for the place of residence, caste, religion, women’s age, age at marriage, parity, women’s education, partner’s education, women’s employment status, household size, wealth index, decision-making on healthcare, access to mass media and region. Unadjusted results show that women who experienced IPV were less likely to receive adequate ANC (Unadjusted OR: 0.63, 95% CI 0.59–0.67), delivery assistance from a skilled health provider (Unadjusted OR: 0.68, 95% CI 0.64–0.73) and PNC within two days of delivery (Unadjusted OR: 0.82, 95% CI 0.77–0.87) as compared to those who did not experience IPV. After adjusting for socio-demographic characteristics, the relationship between IPV and adequate ANC was attenuated, however, remained significant (Adjusted OR: 0.90, 95% CI: 0.84–0.97). We did not find any evidence of an association between women’s exposure to IPV and skilled delivery assistance in the adjusted analysis. Surprisingly, we find that IPV was related to an increased likelihood of receiving PNC in the adjusted analysis (Adjusted OR: 1.09, 95% CI: 1.02–1.16).

**Table 2:** Unadjusted and adjusted odds ratio from binary logistic regression models investigating the relationship between intimate partner violence (IPV) and the utilization of maternal healthcare services, India (NFHS-4).

Variables	Adequate ANC (Four or more ANC visits)		Delivery assistance by skilled health provider		PNC within two days of delivery	
	Unadjusted OR (95% CI)	Adjusted OR (95% CI)	Unadjusted OR (95% CI)	Adjusted OR (95% CI)	Unadjusted OR (95% CI)	Adjusted OR (95% CI)
**Intimate partner violence (Any form of violence)**						
No	1.00	1.00	1.00	1.00	1.00	1.00
Yes	0.63 (0.59–0.67)**	0.90 (0.84–0.97)**	0.68 (0.64–0.73)**	0.98 (0.90–1.06)	0.82 (0.77–0.87)**	1.09 (1.02–1.16)**
**Place of residence**						
Urban		1.00		1.00		1.00
Rural		0.94 (0.87–1.02)		0.89 (0.79–0.99)*		0.99 (0.92–1.07)
**Caste**						
SC/ST		1.00		1.00		1.00
OBC		0.78 (0.72–0.83)**		1.19 (1.09–1.30)**		1.01 (0.94–1.08)
Others		0.97 (0.89–1.06)		1.08 (0.96–1.21)		1.07 (0.98–1.17)
**Religion**						
Hindu		1.00		1.00		1.00
Muslim		1.21 (1.10–1.33)**		0.63 (0.56–0.70)**		0.93 (0.85–1.01)
Others		0.77 (0.69–0.85)**		0.57 (0.50–0.66)**		0.86 (0.77–0.96)**
**Women's age (years)**						
15–24		1.00		1.00		1.00
25–34		1.16 (1.08–1.25)**		1.07 (0.97–1.18)		1.05 (0.97–1.13)
35–49		1.28 (1.13–1.45)**		1.04 (0.90–1.20)		1.07 (0.95–1.20)
**Age at marriage (years)**						
< 18		0.85 (0.79–0.91)**		0.94 (0.87–1.01)		0.87 (0.82–0.93)**
≥ 18		1.00		1.00		1.00
**Parity**						
1		1.00		1.00		1.00
2–3		0.76 (0.71–0.82)**		0.56 (0.50–0.63)**		0.79 (0.73–0.85)**
≥ 4		0.53 (0.47–0.60)**		0.41 (0.36–0.48)**		0.61 (0.55–0.68)**
**Women's education**						
No education		1.00		1.00		1.00
Primary		1.22 (1.10–1.35)**		1.25 (1.12–1.39)**		1.13 (1.03–1.24)**
Secondary		1.42 (1.30–1.56)**		1.57 (1.42–1.74)**		1.19 (1.09–1.29)**
Higher		1.58 (1.37–1.83)**		2.31 (1.79–2.97)**		1.27 (1.10–1.47)**
**Partner's education**						
No education		1.00		1.00		1.00
Primary		1.18 (1.06–1.32)**		1.21 (1.08–1.35)**		1.16 (1.05–1.28)**
Secondary		1.13 (1.03–1.24)**		1.27 (1.15–1.40)**		1.18 (1.08–1.29)**
Higher		1.09 (0.95–1.25)		1.51 (1.24–1.83)**		1.20 (1.05–1.37)**
**Women's employment status**						
Unemployed		1.00		1.00		1.00
Employed		1.08 (1.00–1.17)		0.87 (0.80–0.95)**		1.11 (1.03–1.19)**
**Household size**						
1–4		1.00		1.00		1.00
5–6		0.95 (0.88–1.02)		0.93 (0.85–1.02)		0.99 (0.93–1.06)
≥ 7		0.79 (0.73–0.86)**		0.83 (0.75–0.92)**		0.88 (0.81–0.95)**
**Wealth index**						
Poorest		1.00		1.00		1.00
Poorer		1.27 (1.16–1.40)**		1.37 (1.24–1.50)**		1.19 (1.09–1.29)**
Middle		1.68 (1.51–1.86)**		2.00 (1.77–2.26)**		1.57 (1.42–1.74)**
Richer		2.13 (1.89–2.40)**		3.09 (2.63–3.63)**		1.84 (1.64–2.07)**
Richest		2.86 (2.48–3.30)**		4.83 (3.85–6.06)**		2.50 (2.16–2.89)**
**Decision-making on healthcare**						
Wife alone		1.00		1.00		1.00
Wife & Husband		1.03 (0.93–1.15)		1.00 (0.88–1.14)		1.04 (0.94–1.16)
Husband alone/ others		0.79 (0.71–0.89)**		1.00 (0.87–1.15)		0.88 (0.79–0.98)*
**Access to mass media**						
No		1.00		1.00		1.00
Yes		1.74 (1.60–1.89)**		1.24 (1.14–1.36)**		1.33 (1.23–1.43)**
**Region**						
North		1.00		1.00		1.00
Central		0.64 (0.59–0.70)**		0.83 (0.74–0.93)**		0.92 (0.84–1.01)
East		0.92 (0.83–1.01)		1.07 (0.95–1.21)		0.96 (0.87–1.06)
Northeast		1.03 (0.92–1.15)		0.63 (0.54–0.73)**		0.63 (0.56–0.71)**
West		2.33 (2.06–2.63)**		1.17 (0.99–1.39)		1.08 (0.96–1.22)
South		3.28 (2.90–3.72)**		3.91 (2.04–5.02)**		1.21 (1.08–1.37)**

Significance level: **p ≤ 0.01, *p ≤ 0.05.

Abbreviations: *OR* odds ratio, *CI* confidence interval.

The results also show that women residing in rural areas were less likely to have delivery assistance from a skilled provider (Adjusted OR: 0.89, 95% CI: 0.79–0.99) as compared to urban women. Women who belonged to OBC had lower odds of adequate ANC (Adjusted OR: 0.78, 95% CI: 0.72–0.83) and higher odds of skilled delivery assistance (Adjusted OR: 1.19, 95% CI: 1.09–1.30) than those who belonged to the SC/ST group. Muslim women were associated with an increased likelihood of adequate ANC (Adjusted OR: 1.21, 95% CI: 1.10–1.33) and a lower likelihood of skilled delivery assistance (Adjusted OR: 0.63, 95% CI: 0.56–0.70), as compared to Hindus. Women who married below 18 were less likely to receive adequate ANC (Adjusted OR: 0.85, 95% CI: 0.79–0.91) and PNC within two days (Adjusted OR: 0.87, 95% CI: 0.82–0.93) than those who married at 18 years or older. Educational attainment of both women and partners was found to be positively associated with all three indicators of maternity care services. Employed women had a lower likelihood of receiving skilled delivery assistance (Adjusted OR: 0.87, 95% CI: 0.80–0.95), whereas the odds of PNC use were higher among employed women (Adjusted OR: 1.11, 95% CI: 1.03–1.19), as compared to the unemployed ones. Women residing in large households (seven or above members) were a lower recipient of adequate ANC (Adjusted OR: 0.79, 95% CI: 0.73–0.86), skilled assistance during delivery (Adjusted OR: 0.83, 95% CI: 0.75–0.92), and PNC within two days (Adjusted OR: 0.88, 95% CI: 0.81–0.95). Wealth status had a strong and positive influence on the utilization of maternal healthcare services. The odds of maternity care increased from the bottom to the richest wealth group in all three components of maternal healthcare services. Women whose husbands alone or others make healthcare decisions were significantly less likely to receive adequate ANC (Adjusted OR: 0.79, 95% CI: 0.71–0.89) and PNC within two days (Adjusted OR: 0.88, 95% CI: 0.79–0.98), as compared to women who alone made decisions on healthcare. Women who had access to mass media were associated with an increased likelihood of maternal healthcare utilization. Geographical region variable also had a significant influence on maternity care utilization. Women from the central region were less likely to have adequate ANC, whilst women from the west and south regions had a higher likelihood of adequate ANC. The likelihood of skilled delivery assistance was lower in the central and northeast regions, whereas women who resided in the south region were associated with a higher likelihood of delivery assistance by the skilled provider and PNC within two days of delivery (Table 2).

Furthermore, we disaggregated our analysis to examine the nuanced associations between various forms of IPV and the utilization of maternal healthcare services. The results show that women’s exposure to physical (Adjusted OR: 0.89, 95% CI: 0.83–0.95) and sexual violence (Adjusted OR: 0.84, 95% CI: 0.74–0.95) was significantly associated with a lower likelihood of adequate ANC, even after accounting for socio-demographic covariates. However, emotional violence had no significant relationship with ANC utilization in the adjusted analysis. In regard to the delivery assistance by the skilled health provider, all three forms of IPV were insignificant when we controlled covariates in the adjusted models. Moreover, women who were exposed to physical violence slightly had higher odds (Adjusted OR: 1.08, 95% CI: 1.01–1.16) of PNC within two days of delivery. Sexual and emotional violence had no significant relationship with PNC usage in the adjusted analysis (Table 3).

**Table 3:** Unadjusted and adjusted odds ratio from binary logistic regression models investigating the relationship between women’s exposure to various forms of IPV and the utilization of maternal healthcare services, India (NFHS-4).

Various forms of IPV	Adequate ANC (Four or more ANC visits)		Delivery assistance by skilled health provider		PNC within two days of delivery	
	Unadjusted OR (95% CI)	Adjusted OR (95% CI)	Unadjusted OR (95% CI)	Adjusted OR (95% CI)	Unadjusted OR (95% CI)	Adjusted OR (95% CI)
**Physical violence**						
No	1.00	1.00	1.00	1.00	1.00	1.00
Yes	0.60 (0.56–0.63)**	0.89 (0.83–0.95)**	0.68 (0.63–0.73)**	0.98 (0.90–1.07)	0.81 (0.76–.86)**	1.08 (1.01–1.16)*
**Sexual violence**						
No	1.00	1.00	1.00	1.00	1.00	1.00
Yes	0.54 (0.49–0.61)**	0.84 (0.74–0.95)**	0.63 (0.56–0.71)**	0.94 (0.82–1.08)	0.76 (0.68–0.85)**	1.05 (0.94–1.18)
**Emotional violence**						
No	1.00	1.00	1.00	1.00	1.00	1.00
Yes	0.74 (0.68–0.80)**	0.97 (0.88–1.07)	0.74 (0.67–0.81)**	0.96 (0.86–1.08)	0.81 (0.75–0.88)**	1.01 (0.92–1.11)

Adjusted models were controlled for place of residence, caste, religion, women’s age, age at marriage, parity, women’s education, partner’s education, women’s employment status, HH size, wealth index, decision-making on healthcare, access to mass media, and region.

Significance level: **p ≤ 0.01, *p ≤ 0.05.

Abbreviations: *OR* odds ratio, *CI* confidence interval.

## Discussion

This is one of the first studies to investigate the relationship between women’s experience of IPV and the utilization of maternal healthcare services in India, using a nationwide representative large-scale sample survey. Our study has found that about 26% of the sample women (current married) experienced violence perpetrated by their partners in the past year. Our estimate on the prevalence of IPV is consistent with a study conducted among currently married Indian women^44^. This persistent high prevalence of IPV reflects strong patriarchal gendered norms of society where men are considered to be superior over women^10^.

The present study shows that women’s experience of IPV was associated with 10% lower odds of adequate ANC, after adjusting for relevant socio-demographic covariates. An earlier study conducted in India also reported that physical violence during pregnancy decreased the likelihood of prenatal care^45^. Our finding is also consistent with several previous studies carried out in Bangladesh^28^, Nigeria^46^, Ethiopia^29,33^, and Mozambique^31^. The findings of this current study highlight the urgency of adequate maternal healthcare needs among the women who are the victims of domestic violence. A woman who experienced physical, sexual, or emotional violence may endure powerless to control over sexual and reproductive healthcare, particularly when she required permission from the male partner. Furthermore, domestic violence is negatively correlated with decision-making autonomy in the household and positively related to the controlling behavior of the husband^47–49^, which could impede women from acquiring their required reproductive healthcare services. Given the lack of autonomy in healthcare-seeking, women who experienced domestic violence are likely to feel discouraged in receiving maternal healthcare services. Our study, however, found no significant relationship between women’s experience of IPV and skilled assistance during delivery in the adjusted analysis. Unexpectedly, women’s encounter with IPV was positively correlated with PNC within two days of delivery, after accounting for socio-demographic confounders in the adjusted model. In further exploring the unexpected relationship between women’s exposure to IPV and PNC usage, it is observed that the odds of PNC were attenuated and twisted in the opposite direction once we controlled for socio-demographic factors, in particular wealth status of the household and educational attainment of women. The influence of these two variables (wealth and education) principally altered the association in the adjusted analysis.

We also found considerable differences in the utilization of maternal healthcare services by socio-demographic factors. It is observed that women with higher educational attainment, those from the upper quintiles of household wealth, and having access to mass media were positively associated with the utilization of maternity care services. On the contrary, women with high parity, those who married early (below 18 years) and resided in large households were associated with a decreased likelihood of maternal healthcare utilization. These findings are consistent with several prior studies conducted in India and elsewhere^39,49–52^. Our study also found that women’s non-involvement in decision-making on healthcare was associated with lower use of maternal healthcare which is in accordance with an earlier study^46^. Furthermore, the geographical residence had a significant influence on maternal healthcare-seeking. In agreement with a previous study^50^, the present study also showed that women residing in the southern region were more likely to receive adequate ANC and delivery assistance from a skilled health provider.

The present study is not without limitations. Firstly, we could not draw the causal link between women’s experience of IPV and maternity care due to the cross-sectional nature of the study design. Secondly, the data are self-reported and retrospective. Therefore, the potential recall bias in our study findings cannot be ignored. Thirdly, women often may not disclose their experiences of violence. Therefore, there could be a possibility of under-reporting of violence due to stigmatization, fear, and sensitivity. Finally, the covariates were selected based on previous research and dependent on the information available in the dataset. Therefore, there could be other potential factors that might influence the utilization of maternal healthcare which were not incorporated in our present study. Despite the limitations, our study significantly contributed to the existing literature. Firstly, this is a population-based large-scale nationally representative study. Therefore, the study results can be generalized at the national level and comparable to the findings of other countries. Secondly, this study made an important contribution to the existing literature by assessing the association between women’s experience of IPV and the utilization of maternal healthcare services. Moreover, the inclusion of wide-ranging socio-demographic factors as confounding variables made this study's results robust and resilient. Finally, the evidence of this study provides a unique opportunity to initiate effective interventions for the improvement of maternal healthcare access and utilization among those women who are the victims of domestic violence.

## Conclusion

Our study found that over one-fourth (26%) of sample women experienced violence by their intimate partners in the past year. The findings of this present analysis indicate very strong evidence of a negative relationship between women's experience of IPV and adequate ANC utilization. It is recommended that targeted interventions should be triggered for the healthcare needs of women who are exposed to violence in their intimate relationships. The government should take necessary legal actions against the perpetrator to eliminate violence against women that would improve maternity service utilization and thereby reduce the risk of maternal mortality. Further longitudinal research is needed to explore the potential mechanism mediating the relationship between IPV and utilization of maternal healthcare services.

## Data Availability

The dataset analysed during the current study are available in the Demographic Health Surveys (DHS) repository, https://dhsprogram.com/data/available-datasets.cfm.
